# Programmed system for fatigue life prediction of excavator turntables based on multi-body dynamics and finite element analysis

**DOI:** 10.1016/j.heliyon.2024.e33126

**Published:** 2024-06-15

**Authors:** Chen Xian, Han Zhang, Young-chul Kim, Haochen Zhang, Yantong Liu

**Affiliations:** a School of Mechanical Engineering, Shaanxi Institute of Technology, Xi'an, 710000, PR China; b Department of Mechanical Engineering, Kunsan National University, Gunsan, 54150, Republic of Korea; c Department of Computer Information Engineering, Kunsan National University, Gunsan, 54150, Republic of Korea

**Keywords:** Fatigue prediction, Finite element analysis, Multi-body dynamics modeling, Load spectrum, MATLAB

## Abstract

This study focuses on predicting mechanical fatigue in excavator turntables, critical components susceptible to failure due to variable operational loads. While conventional methods like finite element analysis(FEA) and multiaxial fatigue criteria have been used, they are limited by the complexity and cost of obtaining real operational load spectra. To address this challenge, our research presents a comprehensive approach that integrates multi-body dynamics modeling, finite element analysis, and MATLAB-based fatigue life prediction systems. Our methodology involves creating a finite element model for stress analysis, synthesizing load spectra from operational data, and utilizing Weibull distribution to analyze load magnitude probabilities. Subsequently, MATLAB imported the load spectrum and built the fatigue prediction framework to finalize the analysis. Furthermore, we have fully open-sourced our code on an open platform, incorporating default load profiles and predictive models within the code. Key findings pinpoint areas prone to stress concentration and fatigue. Key findings identify stress concentration areas and fatigue-prone regions, providing valuable insights for design optimization and durability improvement.

## Highlights

• A computational coordinate system using multi-body dynamics and a finite element model in ANSYS were used to analyse the excavator rotary table, using experimentally measured loads to identify critical points and calculate stress and strain.
• We fitted the stress spectra and generated the load spectra using working condition scaling and amplitude density.
• We used MATLAB to create an open-source fatigue prediction method for excavator rotary tables, and open-sourced the entire code and method.

## 1 Introduction

Excavators are crucial engineering machinery extensively utilized in various construction projects, significantly contributing to building construction [[1], [2], [3], [4]].The excavator turntable, a crucial structural component essential for the excavator's operation, supports various machine parts such as the boom, arm, and bucket assembly. It not only bears its own weight but also effectively handles dynamic loads from excavation activities. Additionally, it is highly prone to fatigue fractures [[5], [6], [7], [8]].However, the harsh operating environment and fluctuating loads can cause stress changes, potentially leading to fatigue failure [[9], [10], [11]].

The load spectrum is crucial for assessing fatigue value; however, obtaining an actual load spectrum is complex and costly [[12], [13], [14]].Consequently, many companies lack essential load data, complicating predictions of product fatigue life during design [15,16].Carpinteri et al. [16]studied multiaxial fatigue under varied amplitude and random loading. Wang et al. [17,18] estimated the RCF lifespan of wind turbine carburized gears using a multiaxial fatigue criterion, while Zhu et al. [19]assessed the critical surface criteria of four materials under various loading conditions.Gassne initially proposed the load spectrum concept, which remained undeveloped [20]. Heuler and Klätschke et al. [21] [][21][]explored loading sequence effects on fatigue, offering guidelines for creating new load spectra. Klemenc, Fajdiga, and colleagues et al. [22]applied a hybrid modeling approach to simulate load spectra. Xiong and Shenoi et al. [23] [][23][]developed a method for accelerated load spectra testing, utilizing iso-damage principles to analyze existing load time histories.

The use of numerical methods like the finite element method for estimating fatigue life has gained popularity with advances in simulation technologies. Ural et al. [24] [][24][]explored how the finite element method and fracture mechanics can predict cracks and estimate fatigue life in helicopter bevel gears. Liu et al. [25] [][25][]created a modular approach to study load effects on the efficiency of multirange hydromechanical transmissions. Shinde et al. [26] [][26][]employed a modified rain flow counting method, enabling Miner criterion application for assessing structures' fatigue life under random loading. Mayer et al. [27]examined how cyclic loads below the endurance limit impact fatigue damage amid variable amplitude loads. Medepalli and Rao [28]highlighted the variability in vehicle load characteristics with road conditions and demonstrated the feasibility of predicting road load through numerical simulation.Assuming a constant load fails to accurately depict damage evolution in a fluctuating load spectrum. Liu et al. [29] [][29][]assessed fan blades' fatigue life under random loading, considering stress amplitude and mean stress together. Deng et al. [30] [][30][]explored contact and bending fatigue using the cumulative fatigue criterion and the stress-life equation.

Thus, compiling the load spectrum and choosing damage accumulation rules is critical.Despite advances, practical application gaps remain in using load spectrum analysis for predicting excavator turntable fatigue life. Besides the aforementioned contributions, addressing the challenges and difficulties related to researching load data is crucial, as they greatly affect the prediction of fatigue life in excavator turntables. Despite the crucial role of load spectra in fatigue assessment, acquiring actual load data is frequently complex and expensive. Numerous companies face a shortage of fundamental load data, which complicates the prediction of product fatigue life during the design phase. Current methods for compiling load spectra are constrained, leading to gaps in practical applications attributable to the high costs and complexity associated with obtaining real load data. Furthermore, current models might encounter challenges in accurately capturing the subtleties of variable and random loads, thereby impeding precise fatigue life predictions. By recognizing these challenges, our research endeavors to narrow the chasm between theoretical modeling and real-world operational data, providing a comprehensive and flexible fatigue prediction system tailored for excavator turntables.

These gaps arise from the high costs and complexity of acquiring real load data and the limitations of current models to capture variable and random load nuances.Previous studies indicate that accurately estimating bevel gears' fatigue life requires the load spectrum, mathematical and logical construction of fatigue S–N curves, fatigue damage, and calculations.Current studies often overlook excavator rotary tables, missing an integrated framework for their fatigue prediction.Addressing these issues requires developing an adaptable, integrated fatigue prediction system for various scenarios.

This paper makes the following contributions.

1. A computational coordinate system was established to model and analyze the excavator's rotary table and its components using multi-body dynamics based on the principle of linear superposition.
2. This work established a finite element model of the excavator turntable in ANSYS, applied an experimentally measured external load to identify the turntable's critical points, and calculated the stress and strain values at these points.
3. This work conducted zero-drift processing, peak and valley removal, and other adjustments on the synthetic stress spectrum to synthesize the load spectrum, utilizing the proportion of each working condition and the amplitude's probability density parameter.
4. Utilizing the MATLAB platform, we developed a fatigue life prediction system for the excavator rotary table. All codes were made open-source and uploaded to MATLAB community's open access platform.

## 2 Multi-body dynamics analysis

Hydraulic excavator is a combination of various parts of the mechanical equipment, coupled with the working conditions and the uncertainty of the working environment, so it is necessary to analyze the force of its parts, this chapter is mainly on the excavator turntable and other parts of the working device force analysis.

In this paper, according to Shanhe Intelligent WY350 excavator, the force analysis of each part is carried out. The excavator parameters are shown in Table 1.

**Table 1:** Excavator parameters.

Projects		Parameters
Engine	Model	SWE350ES
	Rated power.kW	183.9 kW
	Rated speed.r/min	2000
Working device	Standard bucket capacity.m³	1.6
	Boom length.mm	6500
	Bucket length.mm	3260
Operating range	Maximum digging radius L1.mm	11300
	Maximum digging height L2.mm	7580
	Maximum digging depth L3.mm	7450
	Maximum unloading height L4.mm	7580
	Maximum vertical digging depth L5.mm	6950
	Maximum digging radius on level ground L6.mm	11300
Performance	Maximum digging force (bucket bar/bucket).kN	163/210

**Fig. 1:**
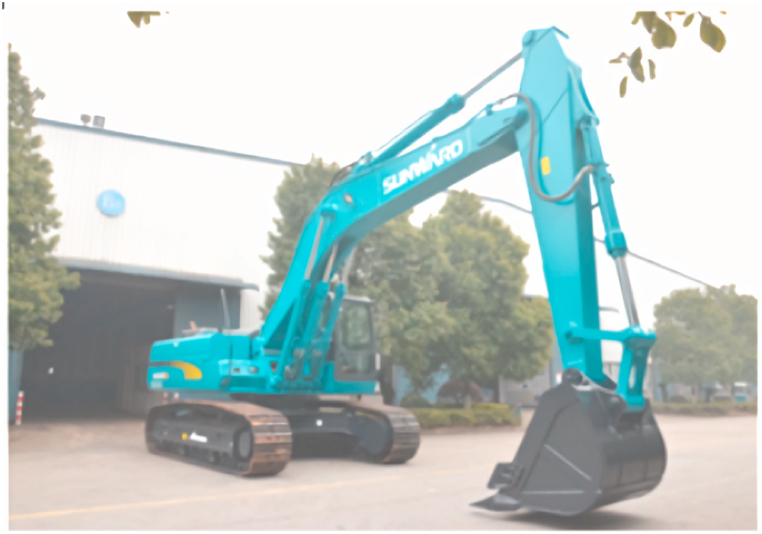
Shanhe intelligent SWE350ES excavator.

As can be seen from Fig. 1, the hydraulic excavator consists of a working device, a turntable, and a traveling device. Hydraulic excavator is very complex, in the work of the need for each part of the coordination, common role, this section of the excavator for the establishment of mathematical models as well as simplification, for the following analysis of the turntable of each articulation point of the force to lay the foundation [31].

**Fig. 2:**
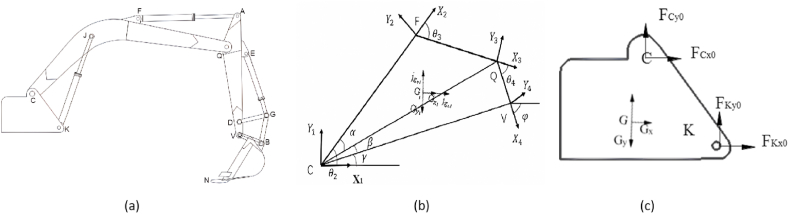
Force analysis process.

Based on the above, the mathematical model of the excavator is established as follows, as shown in Fig. 2(a): point C - the articulation point of the movable arm and the rotary table; point K - the articulation point of the movable arm cylinder and the rotary table; point J - the articulation point of the -Point J - articulation point of moving arm cylinder and moving arm; Point F - articulation point of rocker cylinder and moving arm; Point Q - articulation point of moving arm and rocker arm; Point A - articulation point of rocker cylinder and rocker arm. - articulation point of rocker arm cylinder and rocker arm; Point D - articulation point of bucket cylinder and rocker arm; Point E − articulation point of rocker rod and rocker arm; Point G - articulation point of - the triple articulation point of the remote rod, connecting rod, and bucket cylinder; Point B - the articulation point of the bucket and rocker arm; Point V - the articulation point of the connecting rod and the bucket; Point N - the N - the tip of the bucket teeth [32].

### 2.1 Kinematics positive solution

The force analysis in this paper uses the D-H principle, and the D-H coordinate method [33,34], an efficient computational method proposed by Denavi and Hartenbg, belongs to the category of efficient and accurate modeling methods, which are mainly applied to inverse kinematics modeling in the study of multi-body systems. The motion of an excavator at work can be briefly described as the following two cases: 1. the slewing motion of the excavator's slewing structure; and 2. the rotation of the other components of the working device around the pin driven by its hydraulic cylinders. Considering the working device of the excavator as a four-link structure, the coordinate system of the excavator was established using the D-H principle, aiming at obtaining the working state of the excavator as well as the angle of rotation at any moment through coordinate transformation.

Mechanical modeling is mainly divided into kinematic positive and kinematic inverse solutions, as shown in Fig. 2(b) and (c).

According to the D-H principle, the data related to the working device of the excavator were brought into the matrix to obtain the transformation matrix for the mutual transformation of neighboring coordinate systems as follows:

(1) $$M_{1}^{0}=\left[\begin{array}{cccc} \cos\;\theta_{1} & 0 & \sin\;\theta_{1} & \alpha_{1}\;\cos\;\theta_{1}\\ \sin\;\theta_{1} & 0 & -\cos\;\theta_{1} & \alpha_{1}\;\sin\;\theta_{1}\\ 0 & 1 & 0 & d_{1}\\ 0 & 0 & 0 & 1 \end{array}\right]$$

(2) $$M_{2}^{1}=\left[\begin{array}{cccc} \cos\;\theta_{2} & -\sin\;\theta_{2} & 0 & \alpha_{2}\;\cos\;\theta_{2}\\ \sin\;\theta_{2} & \cos\;\theta_{2} & 0 & \alpha_{2}\;\sin\;\theta_{2}\\ 0 & 0 & 1 & 0\\ 0 & 0 & 0 & 1 \end{array}\right]$$

(3) $$M_{3}^{2}=\left[\begin{array}{cccc} \cos\;\theta_{3} & -\sin\;\theta_{3} & 0 & \alpha_{3}\;\cos\;\theta_{3}\\ \sin\;\theta_{i} & \cos\;\theta_{3} & 0 & \alpha_{3}\;\sin\;\theta_{3}\\ 0 & 0 & 1 & 0\\ 0 & 0 & 0 & 1 \end{array}\right]$$

(4) $$M_{4}^{3}=\left[\begin{array}{cccc} \cos\;\theta_{4} & -\sin\;\theta_{4} & 0 & \alpha_{4}\;\cos\;\theta_{4}\\ \sin\;\theta_{i} & \cos\;\theta_{4} & 0 & \alpha_{4}\;\sin\;\theta_{4}\\ 0 & 0 & 1 & 0\\ 0 & 0 & 0 & 1 \end{array}\right]$$

From the above matrix the transformation matrix of the excavator working device can be obtained as follows:

(5) $$M_{4}^{0}=M_{1}^{0}\times M_{2}^{1}\times M_{3}^{2}\times M_{4}^{3}=\left[\begin{array}{cccc} c_{1}c_{234} & c_{1}s_{234} & s_{1} & c_{1}\left(a_{4}c_{234}+a_{3}c_{23}+a_{2}c_{2}+a_{1}\right)\\ s_{1}c_{234} & s_{1}s_{234} & -c_{1} & s_{1}\left(a_{4}c_{234}+a_{3}c_{23}+a_{2}c_{2}+a_{1}\right)\\ s_{234} & -c_{234} & 0 & a_{4}s_{234}+a_{3}s_{23}+a_{2}s_{2}+d_{1}\\ 0 & 0 & 0 & 1 \end{array}\right]$$

Where, $s_{i}=\sin\;\theta_{i},s_{ij}=\sin\left(\theta_{i}+\theta_{j}\right),s_{ijk}=\sin\left(\theta_{i}+\theta_{j}+\theta_{k}\right)$,

$s_{i}=\cos\;\theta_{i},c_{ij}=\cos\left(\theta_{i}+\theta_{j}\right),c_{ijk}=\cos\left(\theta_{i}+\theta_{j}+\theta_{k}\right)$.In the bucket coordinate system, the coordinates of the bucket tooth tip are set to $P_{4}={\left[0,0,0,1\right]}^{T}$. The vector coordinates in the 0th coordinate system are $P_{0}={\left[X,Y,Z,1\right]}^{T}$, i.e., $P_{0}=M_{4}\times P_{4}$, which is brought into the above equation to obtain the expression for the tooth tip position attitude:

(6) $$\left\{\begin{array}{c} X=c_{1}\left(a_{4}c_{234}+a_{3}c_{23}+a_{2}c_{2}+a_{1}\right)\\ Y=s_{1}\left(a_{4}c_{234}+a_{3}c_{23}+a_{2}c_{2}+a_{1}\right)\\ Z=a_{4}s_{234}+a_{3}s_{23}+a_{2}s_{2}+d\\ \varphi =\theta_{2}+\theta_{3}+\theta_{4} \end{array}\right\}$$

In Equation (6), each length of the excavator's working device is known, *a*_*1*_ is the distance between the origin of the 0th coordinate system and the origin of the first coordinate system 0 in the x-direction, *a*_*2*_ is the distance between the hinge point of the upper frame and the bucket, *a*_*3*_ is the distance between the hinge point of the movable arm and the hinge point of the bucket, *a*_*4*_ is the distance between the bucket and the bucket teeth, and *d*_*i*_ is the distance between the movable arm and the hinge point of the upper frame.

*Φ* is the angle between the bucket tooth tip and the bucket hinge point, which describes the attitude of the bucket. According to the spatial variables $\left[\theta_{1},\theta_{2},\theta_{3},\theta_{4}\right]$ the position and attitude of the bucket teeth can be precisely described, while [x,y,z,φ] describes the position and attitude of the bucket tooth tips.

At this point, the kinematic solution has been solved. Next, we solve the inverse kinematic solution.

### 2.2 Kinematic inverse solution

Similar to the kinematic positive solution, the kinematic inverse solution is solved in reverse according to the D-H principle. That is, the spatial position of the excavator working device and the attitude variable [x,y,z,φ] are known to find out the spatial variable $\left[\theta_{1},\theta_{2},\theta_{3},\theta_{4}\right]$ of the excavator working device. and this spatial variable has and has only one, which can be geometrically related to find out the angle of that turn [35]. Without considering the slewing, taking the 0th coordinate system as the base coordinate system and the hinge point of the movable arm as the coordinate origin, the three-dimensional problem of the excavator working device is transformed into a two-dimensional problem to simplify the analyzing process, which can be assumed that the coordinates of the bucket tooth tip are [x,0,z,φ].

A simplification of the coordinates of the working unit of a hydraulic excavator is shown in Fig. 2(b).

From Fig. 2(b), *θ*_*2*_, *θ*_*3*_, and *θ*_*4*_ can be expressed as respectively:

(7) $$\theta_{2}=\alpha +\beta +\gamma$$

(8) $$\theta_{3}=∠\text{CFQ}-\pi$$

(9) $$\theta_{4}=\varphi -\theta_{2}-\theta_{3}$$

Thus, the coordinates $\left(X_{Q},Z_{Q}\right)=\left(X-QV\;\cos\;\varphi ,Z-QV\;\sin\;\varphi \right)$ of Q can be obtained. In the sketch, α, β are located in triangle CQF and triangle CQV, and according to the cosine theorem, the values of *α*, *β* and CFQ can be obtained as follows:

(10) $$\begin{array}{l} \alpha =\cos^{-1}\frac{CQ^{2}+a_{2}^{2}-a_{3}^{2}}{2CQ•a_{2}}\\ \beta =\cos^{-1}\frac{CQ^{2}+CV^{2}-a_{4}}{2CQ•CV}\\ ∠CFQ=\cos^{-1}\frac{a_{2}^{2}+a_{3}^{2}-CQ^{2}}{2a_{2}a_{3}} \end{array}$$

Meanwhile, the coordinates of point V are known and *γ* is found as follows: $\gamma =\tan^{-1}\frac{z}{x}$.

Taking the slewing platform slewing into account, *α*, *β*, *γ*, and ∠CFQ are independent of the slewing platform slewing and do not change with the base slewing, but at this point the slewing joint angle *θ*_*1*_ is not 0. Express the parameters of the 1st coordinate system in terms of the 0th coordinate system, i.e., the x-coordinate is replaced by $\sqrt{{\left(x-a_{1}\right)}^{2}+y^{2}}$, and the z-coordinate is replaced by z-d1 with the known quantities of $\theta =\tan^{-1}\frac{y}{x}$ for a1, and d1, and the joint angles of rotation are expressed in terms of the final coordinates [x,y,z,φ] as:

(11) $$\left\{ \begin{array}{c} \theta_{1}=\tan^{-1}\frac{y}{x}\\ \theta_{2}=\alpha +\beta +\lambda \\ \theta_{3}=∠CFQ-\pi \\ \theta_{4}=\varphi -\theta_{2}-\theta_{3} \end{array}\right.$$

where: $CQ=\sqrt{{\left(x-a_{4}\;\cos\;\varphi -a_{1}\right)}^{2}+y^{2}+{\left(z-a_{4}\;\sin\;\varphi -d_{1}\right)}^{2}}$, $CV=\sqrt{{\left(x-a_{1}\right)}^{2}+y^{2}+{\left(z-d_{1}\right)}^{2}}$.

The bucket end coordinates [x,y,z,φ] are obtained by geometrical relations with one and only one vector. At the same time, the coordinate angle $\left[\theta_{1},\theta_{2},\theta_{3},\theta_{4}\right]$ of each component in the space of the excavator working device is determined, so that the forward-reverse solution of the angle and the space position attitude is realized.

Up to this point, both forward and reverse kinematic solutions of the excavator have been completed.

### 2.3 Excavator rotary table force analysis

At the outset of analyzing the excavator rotary table's force dynamics, it's critical to understand the foundational principles governing the mechanical interactions at play. The rotary table of an excavator is a pivotal component that undergoes complex forces and moments due to its operational mechanics and structural design. The excavator's slewing platform, essentially the heart of its rotational functionality, is subjected to a variety of forces that dictate its performance and stability. These include gravitational forces, mechanical forces from the movement of the arm and its hydraulic systems, as well as the interaction forces between the platform and the excavator's base frame. Additionally, operational forces such as those generated during the actuation of the rotary mechanism, whether in motion or braking, play a significant role [36].

Fig. 2(c) shows the force analysis of the excavator rotary platform, as shown in the figure, the forces on the slewing platform include.

(1) The combined force G of its own gravity and the gravity of the parts belonging to it.
(2) The force and moment of the moving arm and the hydraulic cylinder of the moving arm and its articulation points C and K. These forces and moments can be applied to the rotary center of the rotary platform in a simplified form, or they can be applied to the corresponding positions according to the specific structural form of the rotary platform or the rotary support.
(3) The force and moment applied to the rotary platform by the base frame through the rotary support.
(4) The rotary driving torque or braking torque generated by the rotary mechanism.

The analysis is carried out in the 0th coordinate system. The slewing platform is subjected to forces and moments at the points C and K where the movable arm and the hydraulic cylinder of the movable arm are articulated with it.

The gravity force of the slewing platform is discretized along the *x*_*0*_ and *y*_*0*_ directions to obtain the gravity component as:

(12) $$G_{x0}=0$$

(13) $$G_{y0}=G$$

The direction of the gravitational component is.

*G*_*x0*_ - Positive direction along axis *x*_*0*_;
*G*_*x0*_ - Negative direction along the axis *y*_*0*_.

Bring the force at the hinge point C into $M_{1}^{0}$, the transformation matrix between the 1st and the 0th coordinate system. Obtain $F_{cx0}$, $F_{cy0}$,and the magnitudes of $M_{cx0}$ and $M_{cy0}$.i.e:

(14) $$F_{cx0}=F_{cx1}\;\cos\;\theta_{2}$$

(15) $$F_{cyx0}=F_{cy1}\;\sin\;\theta_{2}$$

Analyze the force at point K according to the conditions of mechanical equilibrium:

(16) $$\sum F_{Kx0}=0$$

(17) $$\sum F_{Ky0}=0$$

(18) $$F_{Cx0}+F_{Kx0}+Gx0=0$$

(19) $$F_{Cy0}+F_{Ky0}+Gy0=0$$

Up to this point, the hinge points of the slewing platform are sought to be solved.

The data obtained in the experiment are brought into the formula for calculation. The horizontal force at nodes C, K and the vertical force can be found.

**Fig. 3:**
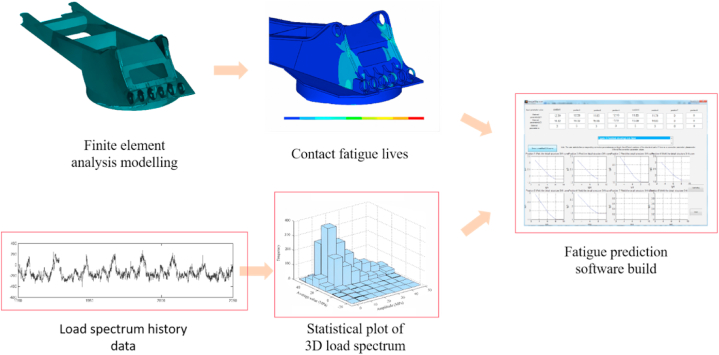
Experimental flow chart.

In summary, all the force analysis required for the experiment has been completed.As Fig. 3 shows the detailed flow of the experiment in detail.

## 3 Model

**Fig. 4:**
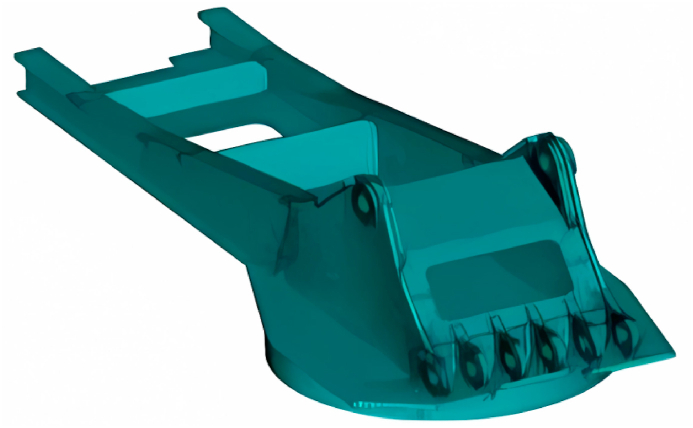
Finite element model of excavator slewing platform.

In this article, the finite element model of the rotary platform is established according to the excavator of Shanhe Intelligence WY350.In this chapter, the 3D software PRO/E is used to establish the 3D model of the slewing support and the upper frame according to the actual dimensions of the excavator of Shanhe Intelligence WY350 in order to carry out the finite element analysis of the rotary platform of the excavator. The aim is to get the stress extreme value position in the model, to carry out the danger point analysis, and to point out the parts that should be strengthened in the design process, as shown in Fig. 4.

### 3.1 Finite element analysis model

The finite element analysis uses the classic analysis software ANSYS, version number: ANSYS 2021 R2. ANSYS [37] has a rich unit library, there are many different units for the user to choose from, the specific selection of which unit type, and the structural form of the model as well as for the model of the computational accuracy has a lot to do with the actual situation of the selection of the most relevant to their We choose the most appropriate unit type according to the actual situation.

**Fig. 5:**
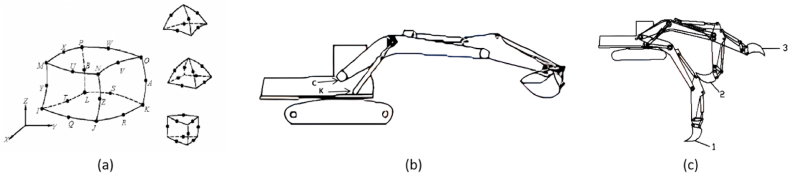
Finite element analysis modeling process.

The excavator slewing platform model established in this chapter includes: excavator upper frame, excavator slewing bearing. Each solid structure are welded, its geometry is more complex, so in this model choose the secondary solid unit Solid186 to establish the model, as far as possible in line with the actual situation, making the calculation more accurate. As Fig. 5(a) Shown demonstrates the Link8 unit detailed process.

After the 3D model is imported into ANSYS, the first step is to determine the material characteristics of the model, which is mainly realized by defining the elastic modulus of the material and Poisson's ratio. The material of the rotary platform of the large excavator is steel, and its material parameters are as follows: density 7850kg/m3, modulus of elasticity 2.06 × 105 MPa, Poisson's ratio 0.29.

Reasonable division of mesh, will make the calculation and analysis more convenient and accurate, so the effective selection of mesh parameters, need to carefully calculate the accuracy and calculation time to determine the degree of mesh thickness.

ANSYS provides users with two different meshing methods: free meshing and mapping. The free meshing method is less controllable, the number and type of cells are not well controlled, and sometimes even the structure of the cell is particularly strange, which will greatly affect the quality of the mesh, affecting the accuracy of the calculation, and increase the computation time is less efficient. The mapping grid precisely makes up for these shortcomings, for large and complex structures, the mapping grid not only improves the computational efficiency, but also improves the computational accuracy, and obtains more accurate analysis results, so this model adopts the mapping grid, so that the number of cells to obtain a better mesh quality, so that the applicability of the stronger. In this chapter, the excavator slewing platform is meshed with SOLID45 cells.

### 3.2 Finite element analysis preliminary work

In this chapter, ANSYS software is used to analyze the rotary table of a large excavator, and before that, it is necessary to determine the calculation working conditions of the excavator. According to the working principle of the excavator rotary table and the working state of the analysis, the calculation of the rotary table in principle choose to make the main beam to produce the maximum bending moment of the working conditions, as follows.

(1) in the maximum digging depth when the bucket digging, while the bucket hydraulic cylinder do move its maximum digging force position, as shown in Fig. 5 (c) in the posture 1 shown. This condition is due to the moving arm hydraulic cylinder force arm is the smallest, from the digging diagram, there may produce the moving arm hydraulic cylinder locking can not be the case, therefore, in Fig. 5 (b) of the C, K two points may produce a lot of force; on the other hand, there is also the possibility of the machine to produce the phenomenon of backward tilting instability, and therefore, the rotary table may be a lot of force and torque.
(2) Critical instability conditions:Critical instability conditions are more, can choose two as a reference: the bucket teeth extend to the furthest end of the ground, so that the excavator reaches the maximum digging radius, at this time, the bucket hydraulic cylinder to do the move, this time the machine is in the critical condition of rearward instability, at this time the machine on the rotary table of the role of the tilting moment is the largest, such as Fig. 5 (c) posture 2 and posture 3, is the whole machine forward tilt instability conditions of the posture, i.e., the moving arm on the lower hinged point of the line is horizontal posture The attitude when the connecting line is horizontal, at this time, let the bucket bar is perpendicular to the stopping surface, and let the bucket to do the action, produce the maximum digging force, so that the excavator is in the forward instability state, but at this time on the rotary table to produce the forward stabilizing torque is the largest.

As shown in Fig. 5 (c), in the maximum unloading radius attitude full bucket slewing and braking, at this time on the rotary table overturning moment, although not very large, but subjected to a large rotary moment of inertia, belonging to the composite load conditions. Excavator modified lifting device and when the lifting moment is the largest, the load of the turntable mainly from the following five aspects.

(1) the self-weight of the components belonging to the turntable.
(2) The force and moment exerted on the rotary table by the movable arm and the force and moment exerted on the rotary table by the articulation point of the hydraulic cylinder of the movable arm.
(3) The load acting on the turntable by the frame through the slewing support, which can be simplified into three kinds of vertical load, overturning moment and radial load.
(4) The load caused by the driving mechanism of the slewing mechanism at the connection with the slewing mechanism.
(5) Inertial load generated by the starting and braking of the rotary table.

The loads applied in the finite element model of the rotary table are.

(1) Gravity of each part. This part of the gravity can be determined according to the specific weight level of the components on the rotary table layout location.
(2) The force of the moving arm and the hydraulic cylinder of the moving arm. They can be applied at the hinge point C and hinge point K as combined forces and moments, respectively, according to the results of the aforementioned force analysis of the working device. In the finite element model, it is still applied to the nodes of the inner peripheral unit of the support in the form of distribution according to the aforementioned method.
(3) The force and moment of the base frame on the rotary table. If you do not consider the slewing bearing, only to study the force and strength of the rotary table, the base frame can be applied to the rotary table axial force, radial force and tilting moment, as well as the circumferential moment generated by the slewing mechanism according to their respective distribution rules applied to the rotary table ring seat ring child nodes of the bottom unit. If the axial loads can be distributed equally on the nodes of the bottom unit of the rotary table, the overturning moments and radial loads can be applied as described in the section on the undercarriage.

In this chapter, only the excavator upper frame, slewing platform, Finite Element Modeling analysis. The following five gradient forces are applied in the horizontal and vertical directions at points C and K, respectively: 100000 N, 400000 N, 700000 N, 1000000 N, 1300000 N.

## 4 Result

### 4.1 Rotary table stress analysis

**Fig. 6:**
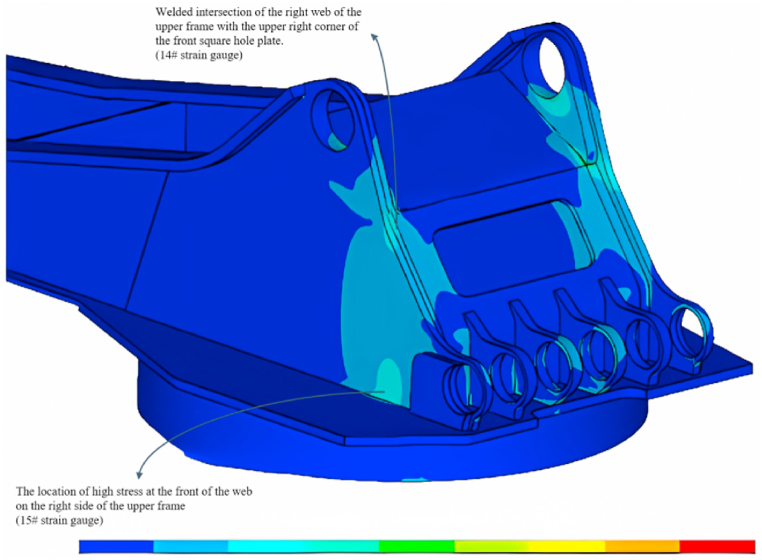
Excavator turntable stress cloud.

In this chapter, ANSYS is used to model and analyze the rotary table of the large excavator, and the stress cloud of the rotary table of the large excavator is obtained, as shown in Fig. 6.

**Fig. 7:**
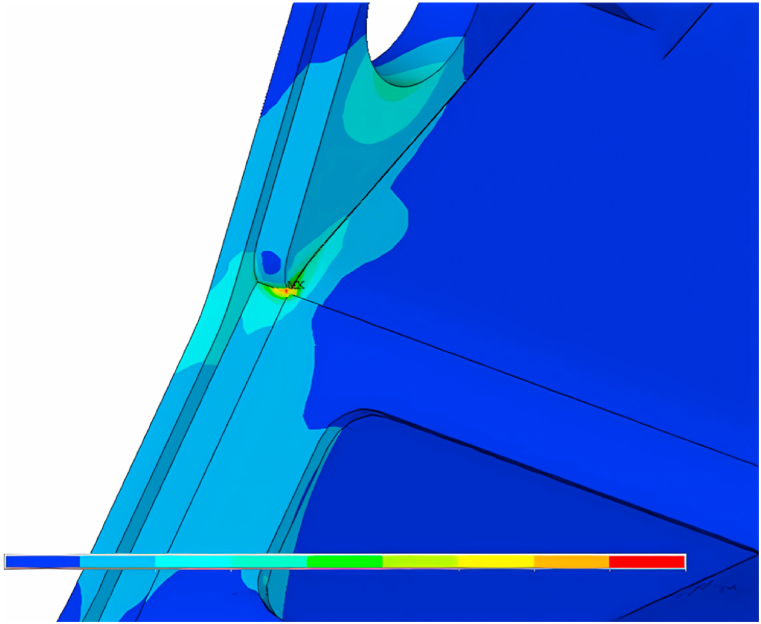
Magnified view of #15 strain gauge.

It can be seen from the stress cloud diagram (see Fig. 7). The stress extreme value is located in the upper frame right side web in the front end of the square hole plate and the upper corner of the intersection of welding (#14 strain gauges) and the upper frame right side web front stress hinge (#15 strain gauges). The stress extreme value is 5 × 1010 MPa, which is due to the stress concentration at the weld.

Danger points C and K, respectively, in the horizontal direction and vertical direction, apply the following five gradient force: 100000 N, 400000 N, 700000 N, 1000000 N, 1300000 N, to get the stress cloud diagram of the excavator turntable. From the stress map, it can be seen that the dangerous point of the excavator is located in the stress extreme value, that is, the right side of the upper frame web in the front end of the square hole plate and the upper corner of the intersection of the welding place (#14 strain gauges) and the right side of the upper frame web in the front end of the stress hinged at the big place (#15 strain gauges).

Take #14 measurement point as an example: according to ANSYS read out the stress measurement point data, get in #14 measurement point measured strain value were: 4.738, 69.706, 134.739, 199.774, 264.810.

The other 7 stress-strain coefficients can be obtained when the horizontal force is applied at #14 and #15 measurement points in the horizontal direction at point C, vertical direction at point C, horizontal direction at point K, and vertical direction at point K, respectively, as shown in Table 2:

**Table 2:** Individual fitting coefficients for #14 and #15 measurement points.

No.	Point C horizontal	Point C vertical	Point K horizontal	Point K vertical
#14 measurement point	4613.900	7023.700	87636.000	−13189.000
#15 measurement point	10064.000	−26045.000	8111.500	−9040.600

According to the principle of force synthesis, the synthesized stresses at measurement point #14 as well as #15 can be obtained from the above table. It is shown below:

(20) $$\delta_{14}=\frac{F_{Ax}}{k_{141}}+\frac{F_{Ay}}{k_{142}}+\frac{F_{Ox}}{k_{143}}+\frac{F_{Oy}}{k_{144}}=\frac{F_{Ax}}{87636}+\frac{F_{Ay}}{-13189}+\frac{F_{Ox}}{4613.9}+\frac{F_{Oy}}{7023.7}$$

(21) $$\delta_{15}=\frac{F_{Ax}}{k_{151}}+\frac{F_{Ay}}{k_{152}}+\frac{F_{Ox}}{k_{153}}+\frac{F_{Oy}}{k_{154}}=\frac{F_{Ax}}{8111.5}+\frac{F_{Ay}}{-9040.6}+\frac{F_{Ox}}{10064}+\frac{F_{Oy}}{-26045}$$

### 4.2 Rotary table load spectrum preparation

First of all, the data in five working conditions, CX (force spectrum in the X direction at point C of the rotary table), CY (force spectrum in the Y direction at point C of the rotary table) and KX (force spectrum in the X direction at point K of the rotary table) and KY (force spectrum in the Y direction at point K of the rotary table), are sorted out into two columns of the time course and the load course respectively in order to prepare for the synthesizing of the externally pushed out stress-load spectrum in Matlab.

The standard variance method is mainly used. The standard variance method is to pre-determine the mean value of the measured data and its variance, and then set the standard deviation multiplier threshold to process the data one by one, when the difference between the processed data and the obtained mean value is greater than the standard deviation multiplier threshold set at the beginning, the data will be excluded, and similarly, when the difference between the processed data and the obtained mean value of the original data is less than the standard deviation multiplier threshold set, the data will be retained.

**Fig. 8:**
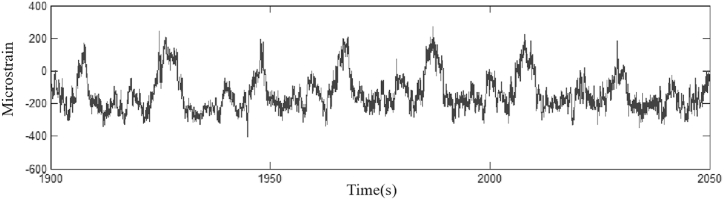
Load spectrum history data.

The data of moderately compacted sandy clay(1800 kg/m³) were used in the experiments, and Fig. 8-Fig. 10 show the working conditions of the experimental results.

Fig. 8 shows the load history data.

**Fig. 9:**
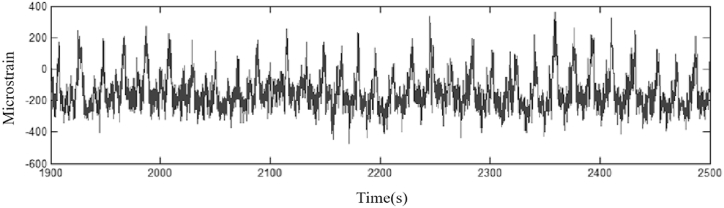
Spectrum of data load before singular value removal.

Fig. 9 shows the load spectrum of the data before singular value removal.

**Fig. 10:**
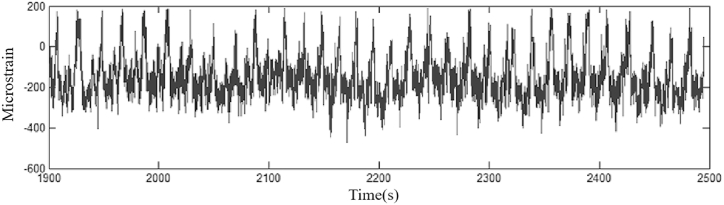
Spectrum of data load after singular value removal.

Fig. 10 shows the data load spectrum after singular value removal.

### 4.3 Symmetric load spectrum

Common fatigue loads are deterministic and random, and there is no simple way to qualitatively and accurately describe the random loads, which can only be described statistically by statistical methods in mathematics. The more widely used are cycle counting method and power spectrum method, rainfall counting method is a statistical method most used in cycle counting method, which retains the effective information of the load spectrum well, making its predicted fatigue cumulative damage similar to the actual situation, and it is widely used in the center of fatigue cumulative damage of the load spectrum technology [38].

**Fig. 11:**
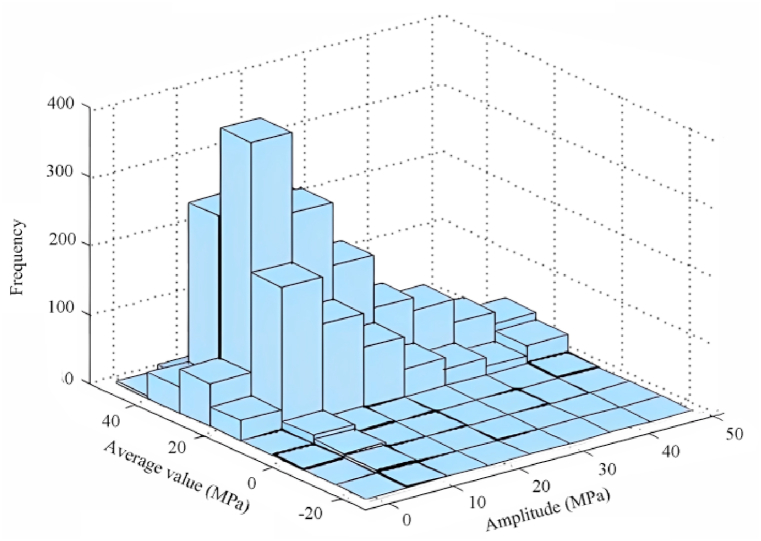
Statistical plot of 3D load spectrum.

The following Fig. 11 shows the three-dimensional load spectrum statistics after Rain flow counting for the load spectrum of hazard point #14 under moderately dense sandy clay (1800 kg/m³).

Excavator rotary tables have a long actual life, mostly belonging to high-cycle fatigue, and they should be predicted for fatigue life using the nominal stress method. In order to obtain more accurate results, the load spectrum is next converted into symmetric cycles in the work in order to carry out the fatigue cumulative damage calculation later.The common methods used in academia are generally the Gerber quadratic curve formula and the Goodman's curve formula.In this paper, the Geber quadratic equation is used to calculate the equivalent magnitude.

The Gerber quadratic curve equation is as follows:

(22) $$S_{eqv}=S_{a}*\frac{\sigma_{b}^{2}}{\sigma_{b}^{2}-S_{m}^{2}}$$

Where $S_{eqv}$ denotes the equivalent magnitude of stress in the part under the premise of equal life; $S_{a}$ denotes the magnitude of stress in the part; $\sigma_{b}$ denotes the limit of tensile strength of the material; and $S_{m}$ denotes the average value of stress in the part.

Based on the above theory and Geber's quadratic formula, a program loop for equivalent symmetric cyclic stress spectra in MATLAB is compiled and the code is resolved as:

*YL11* *=* *getappdata(0,'YLP11′);*

*……*

*str* *=* *get(handles.edit1,'string');*

*JQD* *=* *str2double(str);*

*DXYL11* *=* YL11(:,2).*(JQD^2)./((JQD^2)-YL11(:,1).*YL11(:,1));

*PC11=YL11(:,3);*

*DX11* *=* *[DXYL11,PC11];*

*…‥*.

*DXYL18* *=* YL18(:,2).*(JQD^2)./((JQD^2)-YL18(:,1).*YL18(:,1));

*PC18=YL18(:,3);*

*DX18* *=* *[DXYL18,PC18];*

*set(handles.uitable8,'data',DX18);*

*setappdata(0,'DX11′,DX11);*

*……*

**Fig. 12:**
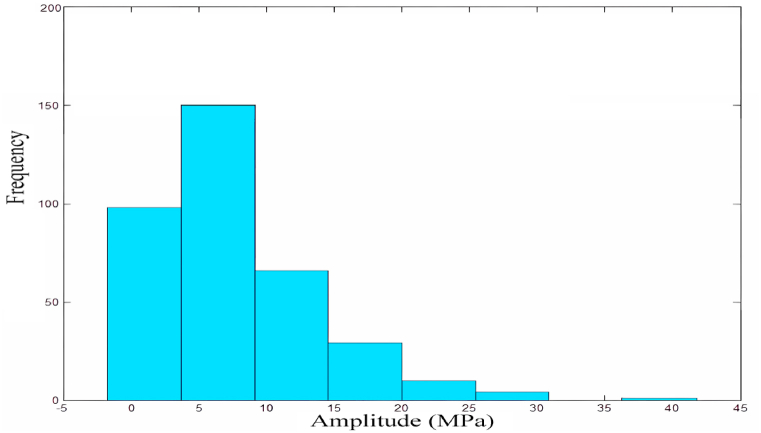
Statistical plot of 2D load spectrum at hazard point #14 after geber transformation.

In order to verify the experimental effect, the experiment transformed the excavator rotary table load spectrum data finally into stress load spectrum data with zero mean value. The results are shown in Fig. 12, which demonstrates the 2D load spectrum statistics of the geber transformed moderately dense sandy clay (1800 kg/m³) at hazard point #14.

### 4.4 Probability density distribution function of load magnitude

According to previous experimental records [39], the load magnitude generally conforms to a Weibull distribution with the following probability density function for the magnitude:

(23) $$f\left(x\right)=b*a^{\left(-b\right)}*{\left(x-c\right)}^{\left(b-1\right)}*\exp\left(-\frac{{\left(x-c\right)}^{b}}{a^{b}}\right)$$

The probability distribution function of the magnitude is as follows:

(24) $$F\left(x\right)=1-\exp\left\{-{\left[\left(x-c\right)/b\right]}^{a}\right\}$$

**Fig. 13:**
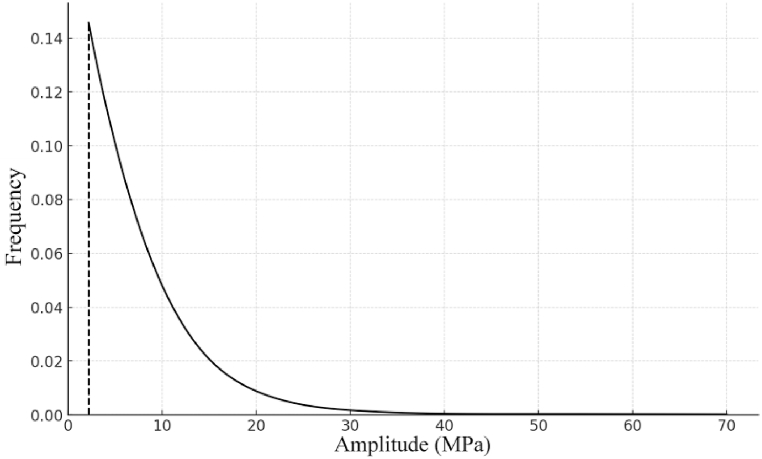
Probability density function curve.

Where *a*: scale parameter, *b*: shape parameter, *c*: position parameter, when the value of *b* is different, the curve shows a normal distribution curve or an exponential distribution curve. When *b* is between *2* and *4*, the density curve approximates a normal distribution. In this paper, the Weibull distribution parameter estimation is used and programmed to be fitted in MATLAB.Fig. 13 shows the amplitude probability density function curve of the load spectrum of the #14 hazardous point of moderately dense sandy clay (1800 kg/m³) fitted by Maximum Likelihood Estimation.Fig. 13 shows that the stress amplitude in the rotary table of this excavator is between 0 and 70 MPa.

### 4.5 Determination of experimental load spectrum

The load spectrum used in the experiment is stepped, because the continuous cumulative frequency curve is difficult to achieve in the experimental loading, so it is necessary to divide the cumulative damage curve into stepped, divided into a variety of levels, this paper adopts the commonly used 8-level load spectrum division [40]. The division coefficients, as well as the magnitude of the magnitude at all levels are shown in Table 3 and Table 4:

**Table 3:** Number of levels and magnitude of levels at Hazard Point #14.

Levels	1	2	3	4	5	6	7	8
Coefficient	1	0.95	0.85	0.725	0.575	0.425	0.275	0.125
Amplitude at each level	69.10786	65.64575	58.73567	50.09807	39.73295	29.36783	19.00272	8.637598

**Table 4:** Number of levels and magnitude of levels at Hazard Point #15.

Levels	1	2	3	4	5	6	7	8
Coefficient	1	0.95	0.85	0.725	0.575	0.425	0.275	0.125
Amplitude at each level	58.16232	55.2542	49.43796	42.16768	33.44333	24.71898	15.99464	7.270288

Table 3 shows the number of levels and magnitude of each level at Hazard Point #14.

Table 4 shows the number of levels and magnitude of each level at Hazard Point #15.

According to the Weibull distribution of the load spectrum of the excavator rotary table, and its amplitude probability density function, the amplitude of the probability density function is applied to integrate, and multiplied by the total frequency to find the frequency of each level and the cumulative frequency, as shown in Table 5 and Table 6.

**Table 5:** Frequency of levels and cumulative frequency of Hazard Point #14.

Levels	1	2	3	4	5	6	7	8
Frequency (each level)	260	343	958	3496	13381	49365	182101	750096
Frequency (Cumulative)	260	603	1561	5057	18438	67803	249904	1000000

**Table 6:** Frequency of levels and cumulative frequency of Hazard Point #15.

Levels	1	2	3	4	5	6	7	8
Frequency (each level)	187	265	776	2951	11788	45322	175265	763446
Frequency (Cumulative)	187	452	1228	4179	15967	61289	236554	1000000

The following is a part of the program in MATLAB to program the number of load cycles at all levels:

*for i* *=* *1:GKS*

*data* *=* *E{i,1}; wblcs* *=* *WBcanshu{i,1};*

*a* *=* *wblcs(1);b* *=* *wblcs(2);c* *=* *wblcs(3);*

*Smax(i,1)* *=* abs(c+a*((-log(1./nwt(i,1)))^(1/b))); datamax(i) *=* max*(data);*

*End*.

*……*

*for n* *=* *1:7*

*zaihepu(n,4)* *=* xishuy(n)*jizhi; xxx *=* *zaihepu(n,4);*

*zaihepu(n,5)* *=* exp*(-((xxx-c)/a).^b); pinlv* *=* *zaihepu(n,5);*

*zaihepu(n,6)* *=* Nk*pinlv;

*end*.

*……*

*for w* *=* *1:8*.

*Fh(w,1)* *=* *w; Fh(w,2)* *=* *xishux(w); Fh(w,3)* *=* xishux(w)*jizhi;

*end*.

*Fh(:,4)* *=* *eee;*

*Fh(:,5)* *=* *ddd;*

*End*.

The final step in making the load spectrum is to set the lifetime represented by a load block to be 1000 h, expand the already expanded frequencies again with the following expansion formula:

(25) $$S_{i}=\frac{nwt_{i}}{n_{i}}*t_{i}*\frac{30}{3600}$$

(26) $$Pc_{i}=\frac{1000}{\left(s_{1}+s_{2}+s_{3}+s_{4}+s_{5}\right)}*eee_{i}$$

Where *nwt*_*i*_ is the number of cycles after the expansion of each condition; *n*_*i*_ is the measured number of cycles for each condition; *t*_*i*_ is the measured number of effective buckets for each condition; *eee*_*i*_ is the number of cycles for each level; *Pc*_*i*_ is the final synthesized extrapolated frequency for each level.

And the final load spectrum is formed, and the final data are shown in Table 7, Table 8.

**Table 7:** #14 hazard point experimental load spectra.

Amplitudes of each level	Mean values of each level	Frequencies of each level	Experimental extrapolation frequency	After rounding
69.10078592	0	260	423.9187874	424
65.64574663	0	343	559.246708	559
58.73566804	0	958	1561.977686	1562
50.0980698	0	3496	5700.077233	5700
39.73295191	0	13381	21817.14344	21817
29.36783402	0	49365	80487.50361	80488
19.00271614	0	182101	296907.8273	296908
8.637598241	0	750096	1222999.18	1222999

**Table 8:** #15 hazard point experimental load spectra.

Amplitudes of each level	Mean values of each level	Frequencies of each level	Experimental extrapolation frequency	After rounding
58.16231684	0	187	288.0227135	288
55.2542	0	265	408.1605299	408
49.43796	0	776	1195.21725	1195
42.16768	0	2951	4545.214051	4545
33.443332	0	11788	18156.21255	18156
24.718984	0	45322	69806.23221	69806
15.994636	0	175265	269948.1331	269948
7.270288	0	763446	1175881.222	1175881

Table 7 shows the experimental load spectrum at hazard point #14, and Table 8 shows the experimental load spectrum at hazard point #15.

## 5 Discussion

This study offers a detailed analysis of fatigue behavior and stress distribution in excavator turntables. Using the Finite Element Modeling (FEM) method, grounded in mechanical analysis and ANSYS simulations, it highlights the critical stress concentration areas, particularly in the right web and welded intersections of the upper frame.Utilizing the developed FEM model, alongside experimentally measured loads and multi-body dynamic analysis, we identified stress distribution and critical points susceptible to fatigue failure. Consequently, we compiled experimental load spectra for the upper frame's right web and welded intersections (designated as parts #14 and #15), based on experiment results.

Table 7, Table 8 present the experimental load spectra for two distinct hazard points on the excavator turntable, among other findings:

**Amplitudes of each level:** These amplitude data are derived from experimental operational data and simulation processes, this column shows the stress amplitude levels experienced at the hazardous points under experimental conditions.

**Mean values of each level:** Indicating that the load spectrum is centered on the mean value of 0, which is typical of alternating stress conditions, where the load alternates from positive to negative values, reflecting the cyclic nature of the loads experienced by the excavator rotary table.

**Frequencies of each level:** It shows the number of cycles (repetitions) of each stress level during the test, simulation. It indicates how often each stress amplitude level occurs and provides insight into the operating load pattern of the excavator.

**Experimental extrapolation frequency:** These values are calculated for each level and can be adjusted for different experimental or operational assumptions to predict how these frequencies will be extrapolated under extended operating conditions. This extrapolation helps to understand the long-term behavior of materials and structures beyond immediate experimental data.

**After rounding:** The last column shows the extrapolated frequencies rounded to the nearest whole number, which simplifies the data for further analysis, such as fatigue life prediction models.

These tables offer valuable insights into the fatigue life and structural integrity of excavator rotary tables under various loading conditions. They can aid future projects in predicting potential failure points and enhancing design for longer service life and improved safety. A hybrid approach combining experimental data and theoretical modeling presents a novel solution to the challenges of acquiring real-world operational data.This paper calculated the load magnitude's probability density function using the Weibull distribution, which accurately reflects real-world conditions. MATLAB was then employed to import the computed data and conduct fatigue life prediction, creating a comprehensive fatigue prediction framework. This framework showcases the potential to enhance the predictive accuracy of engineering analysis.

Additionally, we have open-sourced our code and published it on an open platform.

## 6 Conclusions

This study proposed a multifaceted approach to mechanical fatigue prediction. Initially, we conducted a detailed mechanical analysis of the excavator and its rotary table using various analytical tools. Subsequently, we employed ANSYS Finite Element Modeling (FEM) for modeling and generated the corresponding load spectra. Finally, we imported all data into MATLAB, where we wrote the program's code to finalize the comprehensive mechanical analysis and stress distribution of the rotary table, including an in-depth study of its fatigue behavior and stress distribution.

The study's results identified critical stress concentration areas in the excavator's slewing platform, with a detailed analysis of fatigue failure susceptibility, particularly in the right web of the upper frame and welded intersections.Combining experimental load data with multi-body dynamics and developing experimental load spectra for excavator rotary platforms refined our ability to predict and mitigate potential weaknesses in these engineering structures.

This research introduces a cost-effective method for overcoming challenges in obtaining real-world operational load data and predicting fatigue by merging experimental data with theoretical modeling. We have open-sourced all MATLAB code to aid in predicting the fatigue life of engineering components.The code includes the load spectrum for key components of a large excavator (SWE350) with a maximum digging force of 210 KN and a medium-sized prototype (XE215G) with a maximum digging force of 138 KN. Users can leverage this spectrum to tailor fatigue life predictions to their excavator's maximum digging force.The user interface imposes no model limitations, allowing users to input their stress or force spectrum for life prediction. And made the following contributions.

1. Multifaceted Approach to Fatigue Prediction: This study introduces a comprehensive approach for predicting mechanical fatigue, integrating detailed mechanical analysis, finite element modeling, and MATLAB programming. By integrating these techniques, we obtained insights into stress distribution and fatigue behavior in the excavator's rotary table.
2. Identification of Critical Stress Areas: Our findings pinpoint critical stress concentration areas in the excavator's slewing platform, particularly emphasizing the right web of the upper frame and welded intersections. This detailed analysis improves our comprehension of fatigue failure susceptibility in these engineering structures.
3. Refinement of Predictive Capabilities: By integrating experimental load data with multi-body dynamics and developing experimental load spectra, we have improved our ability to predict and address potential weaknesses in excavator rotary platforms. This cost-effective method addresses challenges in obtaining real-world operational load data and enhances fatigue prediction accuracy.
4. Open-Source MATLAB Code: To promote further research and application, we have made all MATLAB code for predicting the fatigue life of engineering components open-source. This includes load spectra for key excavator components, enabling users to customize predictions based on their specific equipment parameters without any model constraints.

Ongoing development of simulation tools and methods will enhance the accuracy of future models. Publishing our methods and results on an open access platform fosters innovation and sharing of best practices in the engineering community.Future efforts will focus on integrating machine learning and artificial intelligence into fatigue life prediction models, promising to revolutionize engineered structure analysis.

## Funding

This research was supported by the 14th Five-Year Plan Fund for Educational Science in Shaanxi Province (No. SGKCSZ2020871), with the support and assistance of 10.13039/501100002522Kunsan National University.

## Institutional review board statement

Not applicable.

## Data availability

Data will be made available on request.

## Code hosting site

https://matlab.mathworks.com/open/fileexchange/v1?id=161056.

## CRediT authorship contribution statement

**Chen Xian:** Writing – review & editing. **Han Zhang:** Conceptualization. **Young-chul Kim:** Project administration, Conceptualization. **Haochen Zhang:** Project administration. **Yantong Liu:** Writing – review & editing, Software, Funding acquisition.

## Declaration of competing interest

The authors declare the following financial interests/personal relationships which may be considered as potential competing interests: Yantong Liu reports financial support was provided by 10.13039/501100002522Kunsan National University. Chen Xian reports financial support was provided by Shaanxi Province Department of Science and Technology. Young-chul Kim reports financial support was provided by 10.13039/501100002522Kunsan National University Department of Materials Science and Engineering. If there are other authors, they declare that they have no known competing financial interests or personal relationships that could have appeared to influence the work reported in this paper.
